# Homelessness and Maternal and Infant Health

**DOI:** 10.1001/jamanetworkopen.2024.42596

**Published:** 2024-11-06

**Authors:** Mark E. McGovern, Dan Treglia, Erica L. Eliason, Amanda Spishak-Thomas, Joel C. Cantor

**Affiliations:** 1 Rutgers School of Public Health, Rutgers University, Piscataway, New Jersey; 2 Center for State Health Policy, Rutgers University, New Brunswick, New Jersey; 3 Brown University School of Public Health, Providence, Rhode Island

## Abstract

This cross-sectional analysis of pregnancy risk data from US individuals with live births analyzes the association of homelessness with utilization of primary care, rates of stress-induced health behaviors, and allostatic load and anxiety.

## Introduction

Homelessness has significant implications for maternal, infant, and child health (MCH) and health inequities.^1^ Studies using *International Classification of Diseases* codes indicate that the number of postpartum people recorded as being affected by homelessness at the time of delivery is increasing over time.^2^ A multistate study using self-reported housing status demonstrated that experiencing homelessness is associated with behaviors known to affect pregnancy health (eg, smoking), but used data from 2000 to 2007.^3^ We hypothesized homelessness affects MCH through 3 main mechanisms: reduced use of primary care due to residential instability and elevated barriers to routine medical services; higher rates of stress-induced health behaviors such as smoking; and elevated allostatic load and anxiety. In this analysis, we examined associations between homelessness and MCH among samples weighted to be representative of postpartum people in 25 states and New York City from 2016 to 2021.

## Methods

In this cross-sectional study, we used data from the Pregnancy Risk Assessment Monitoring System (PRAMS),^4^ a multi-state cross-sectional survey designed to be representative of individuals with live births. Through mail and telephone questionnaires, and linked birth certificate data, PRAMS collects information on health, sociodemographic characteristics, and behavior. We pooled PRAMS phase 8 datasets from all states asking whether postpartum people were “homeless or had to sleep outside, in a car, or in a shelter” in the year before delivery (eAppendix and eTable 1 in Supplement 1). Informed consent was not required because it was implied by respondents’ survey completion, and as a secondary analysis of deidentified data available from the Centers for Disease Control and Prevention this analysis was deemed not to be human participant research and to be exempt from full review under Rutgers institutional review board. Reporting was guided by the Strengthening the Reporting of Observational Studies in Epidemiology (STROBE) reporting guidelines for cross-sectional studies.

We assessed associations between homelessness and MCH in bivariate analyses and calculated relative risk ratios adjusting for potential confounders using regression models (eMethods in Supplement 1).^5^ Estimates incorporated PRAMS analysis weights, and confidence intervals accounted for sampling (eTable 2 in Supplement 1).

## Results

In a weighted sample of 146 943 postpartum people representing a population of 8 249 272, 2.4% (95% CI 2.3%-2.5%) reported homelessness in the year before birth. Compared with those without homelessness experiences, postpartum people who experienced homelessness were more likely to report their race as Black (34.0% [95% CI, 31.8%-36.2%] vs 14.9% [95% CI, 14.7%-15.1%]), be unmarried, and have high school education or less (Table).

**Table.** Descriptive Statistics by Experience of Homelessness, 2016-2021

Binary and categorical variables	Proportion (95% CI)	
	Did not experience homelessness	Experienced homelessness
Infant is a singleton		
No	0.018 (0.017-0.019)	0.018 (0.014-0.022)
Yes	0.982 (0.981-0.983)	0.982 (0.978-0.986)
Postpartum person is married^a^		
No	0.367 (0.363-0.371)	0.810 (0.791-0.828)
Yes	0.633 (0.629-0.637)	0.190 (0.172-0.209)
Infant sex		
Male	0.490 (0.487-0.494)	0.503 (0.479-0.527)
Female	0.510 (0.506-0.513)	0.497 (0.473-0.521)
Postpartum person’s place of residence		
Rural	0.159 (0.156-0.162)	0.147 (0.131-0.164)
Urban	0.830 (0.827-0.832)	0.842 (0.825-0.858)
Unknown	0.012 (0.011-0.012)	0.011 (0.009-0.013)
Postpartum person’s education^a^		
High school or less	0.349 (0.345-0.353)	0.658 (0.635-0.680)
More than high school	0.642 (0.638-0.646)	0.329 (0.307-0.352)
Unknown	0.009 (0.008-0.010)	0.013 (0.009-0.020)
Postpartum person’s ethnicity^b^		
Not Hispanic	0.840 (0.837-0.843)	0.822 (0.803-0.840)
Hispanic	0.149 (0.146-0.151)	0.163 (0.146-0.182)
Unknown	0.011 (0.010-0.012)	0.014 (0.010-0.022)
Postpartum person’s race^a^		
American Indian	0.004 (0.004-0.005)	0.015 (0.012-0.019)
Asian	0.053 (0.052-0.055)	0.010 (0.007-0.016)
Black	0.149 (0.147-0.151)	0.340 (0.318-0.362)
Hawaiian or Pacific Islander	0.002 (0.002-0.002)	0.002 (0.001-0.005)
White	0.699 (0.696-0.702)	0.514 (0.489-0.538)
Other	0.075 (0.073-0.077)	0.102 (0.089-0.118)
Unknown	0.018 (0.017-0.018)	0.017 (0.012-0.023)
Postpartum person’s partner race^a^		
American Indian	0.004 (0.003-0.004)	0.004 (0.003-0.006)
Asian	0.046 (0.045-0.048)	0.008 (0.005-0.014)
Black	0.120 (0.118-0.123)	0.191 (0.174-0.210)
Hawaiian or Pacific Islander	0.002 (0.001-0.002)	0.002 (0.001-0.004)
White	0.632 (0.629-0.636)	0.281 (0.259-0.304)
Other	0.066 (0.064-0.068)	0.063 (0.052-0.077)
Unknown or not applicable	0.130 (0.127-0.132)	0.451 (0.427-0.475)
Infant is small for gestational age^a^		
No	0.905 (0.903-0.907)	0.867 (0.850-0.882)
Yes	0.095 (0.093-0.097)	0.133 (0.118-0.150)
Postpartum person had postpartum depression^a^		
No	0.877 (0.874-0.879)	0.687 (0.663-0.709)
Yes	0.123 (0.121-0.126)	0.313 (0.291-0.337)
Postpartum person had diabetes during pregnancy^a^		
No	0.903 (0.901-0.905)	0.881 (0.865-0.896)
Yes	0.097 (0.095-0.099)	0.119 (0.104-0.135)
Postpartum person had high blood pressure during pregnancy^a^		
No	0.865 (0.862-0.867)	0.801 (0.782-0.820)
Yes	0.135 (0.133-0.138)	0.199 (0.180-0.218)
**Continuous variables, mean (95% CI)**		
Pregnancy month of first prenatal checkup (1-9)^a^	2.693 (2.680-2.705)	3.533 (3.426-3.641)
Postpartum person’s age^a^	29.340 (29.297-29.383)	26.739 (26.471-27.007)

^a^ *P* < .01.

^b^ *P* < .10.

Homelessness was negatively associated with MCH even when adjusting for observed potential confounders. Adjusted risk ratios were 1.16 (95% CI, 1.12-1.20) for the pregnancy month of the first prenatal checkup, 1.21 (95% CI, 1.09-1.35) for high blood pressure during pregnancy, 1.36 (95% CI, 1.18-1.56) for diabetes during pregnancy, 1.83 (95% CI, 1.68-1.99) for postpartum depression, and 1.17 (95% CI, 1.03-1.34) for small for gestational age (Figure).

**Figure.**
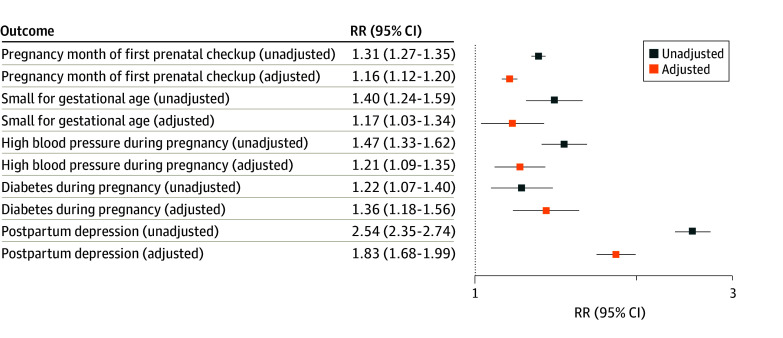
Experience of Homelessness and Maternal and Infant Health in PRAMS Data, 2016-2021 PRAMS indicates Pregnancy Risk Assessment Monitoring System; RR, risk ratio. All data are weighted with the PRAMS survey weights and CIs account for survey design. Analysis is based on PRAMS phase 8. Models for unadjusted relative RRs include only the exposure (an indicator for experience of homelessness) and the outcome. Models for adjusted relative RRs that account for observed potential confounders additionally include the following covariates: infant is a singleton, infant sex, the postpartum person is married, postpartum person’s place of residence (urban or rural), postpartum person’s ethnicity, postpartum person’s race, postpartum person’s partner race, and postpartum person’s age. The month of pregnancy during which the postpartum person had their first prenatal check-up is a count variable ranging from 1 (first month of pregnancy) to 9 (last month of pregnancy), while the other variables are dichotomous indictors. Small for gestational age is defined as a birthweight of less than 10th percentile for gestational age.

## Discussion

This cross-sectional analysis found associations between homelessness and MCH. While PRAMS data are only representative of included states, applying the 2.4% homelessness rate to all US births in 2023 implies 70 000 babies would be born within 12 months of maternal homelessness. We expect increasing numbers of postpartum people to be affected, and concomitant inequities to widen, with expiration of pandemic-era renter protections.^6^ Limitations of this study are that PRAMS is cross-sectional and observational; we were unable to follow up affected populations over time or address potential unmeasured confounding. To the extent that homelessness also raises the risk of stillbirth or miscarriage, estimates focusing on live births may understate its impact on MCH. Self-reports of homelessness may also understate population prevalence. Longitudinal analysis is urgently needed to identify how health evolves over pregnancy, and whether supportive interventions (such as additional prenatal care) can improve MCH among postpartum people who have experienced homelessness and reduce racial and ethnic inequities.

## Notes

Supplement 1. **eAppendix.** Data Source and Sample **eTable 1.** Sample Size by Site, 2016-2021 **eTable 2.** Weighted Response Rates by Site, 2016-2021
eMethods.

eReferences.

Supplement 2.
Data Sharing Statement
