# Supplementary material for: Homelessness and Maternal and Infant Health
Source: JAMA Netw Open. 2024 Nov 6;7(11):e2442596. doi: 10.1001/jamanetworkopen.2024.42596 (PMC11541637; doi:10.1001/jamanetworkopen.2024.42596)
Supplement: Supplement 1. — eAppendix. Data Source and Sample eTable 1. Sample Size by Site, 2016-2021 eTable 2. Weighted Response Rates by Site, 2016-2021 eMethods. eReferences. [file jamanetwopen-e2442596-s001.pdf]

Supplemental Online Content

McGovern M, Treglia D, Eliason EL, Spishak-Thomas A, Cantor JC. Homelessness and maternal and infant health. *JAMA Netw Open.* 2024;7(11):e2442596. doi:10.1001/jamanetworkopen.2024.42596

eAppendix. Data Source and Sample

eTable 1. Sample Size by Site, 2016-2021

eTable 2. Weighted Response Rates by Site, 2016-2021

eMethods

eReferences

This supplemental material has been provided by the authors to give readers additional information about their work.

© 2024 McGovern M et al. *JAMA Network Open.*

eAppendix. Data Source and Sample

In this paper, we used data from the Pregnancy Risk Assessment Monitoring System (PRAMS), which are made available to researchers by the Centers for Disease Control and Prevention (https://www.cdc.gov/prams/php/data-research/index.html). PRAMS is an on-going state-based surveillance system, implemented by state health departments and coordinated by the CDC, that samples postpartum people from birth certificate records (typically 2–6 months after childbirth), and collects data about their experiences before, during, and after pregnancy.[1] Further information about the PRAMS design and methodology are provided in Shulman et al (2018).[2] We used data from the Phase 8 PRAMS Automated Research File, which includes all states that met the minimum survey response rate thresholds for data release. These minimum response rates have changed over time (for example, 55% from 2015-2017 and 50% from 2018-2021), and in this paper we included states that met the response thresholds that applied in their survey year. While PRAMS is primarily state-based, New York City is a separate site from the rest of New York state with its own survey. Therefore, our analysis included 25 states but 26 sites. Puerto Rico and Washington D.C. are also PRAMS sites, but they were not included in this analysis. Given the focus of this paper, we defined our analysis sample as postpartum people who were surveyed about experiencing homelessness. Specifically, we used the following questionnaire item to define our exposure, where postpartum people were asked about events they may have experienced during the 12 months before their new baby was born: "I was homeless or had to sleep outside, in a car, or in a shelter". Respondents answered yes or no to this question. Not all sites included this item in their questionnaire as it was not a core question in Phase 8. Sample sizes responding to this question by site and year are shown in Table A1, and for the relevant sites and years, the weighted response rates in Table A2.

© 2024 McGovern M et al. *JAMA Network Open.*

eTable 1. Sample Size by Site, 2016-2021

	Year of birth						
Site	2016	2017	2018	2019	2020	2021	Total
Alabama		867	792	786	628	683	3,756
Alaska	1,159	1,033	998	1,050	1,008		5,248
Colorado	1,617	1,124	1,117	1,097	1,038	1,252	7,245
Delaware	923	875	837	924	843	827	5,229
Georgia		943	763	781	687	781	3,955
Illinois	1,376	1,219	1,305	1,224	1,198	1,115	7,437
Indiana			858				858
Iowa	966	1,014	827	827	701		4,335
Kansas		986	966	1,001	1,167	1,126	5,246
Kentucky		693	748	903	791		3,135
Louisiana	879	885	844	1,073	693	665	5,039
Maine	812	861	815	816	885	783	4,972
Massachusetts	1,286	1,397	1,412	1,671	1,382	1,303	8,451
Michigan	1,774	1,869	1,844	1,377	1,377	1,355	9,596
Minnesota			1,270	1,050	618	630	3,568
Mississippi			1,183	1,247	998	863	4,291
Missouri	1,053	1,104	921	1,591	958	823	6,450
Nebraska	1,289		1,314	1,598	1,475	1,202	6,878
New York	942	836	762	759		859	4,158
New York City	1,406	1,254	1,480	1,216	1,173	1,237	7,766
Oregon			1,588	2,416	1,656	1,851	7,511
Pennsylvania	1,037	1,216	954	1,119	1,183	919	6,428
Utah	1,392	1,431	1,240	1,703	1,403	1,241	8,410
Washington	1,262	1,230	1,138	1,177	1,232	1,132	7,171
Wisconsin	1,213	1,296	1,026	828	1,659	750	6,772
Wyoming	609	495	543	488	459	444	3,038
Total	20,995	22,628	27,545	28,722	25,212	21,841	146,943

© 2024 McGovern M et al. *JAMA Network Open.*

eTable 2. Weighted Response Rates by Site, 2016-2021

	Year of birth					
Site	2016	2017	2018	2019	2020	2021
Alabama		55	54	56	55	50
Alaska	61	62	60	58	54	
Colorado	59	63	62	59	63	60
Delaware	64	62	59	57	54	52
Georgia		68	59	61	53	50
Illinois	60	55	61	59	61	56
Indiana			51			
Iowa	63	62	51	50	51	
Kansas		63	61	63	66	63
Kentucky		59	60	60	60	
Louisiana	63	66	64	57	57	57
Maine	56	62	57	61	55	50
Massachusetts	60	62	62	61	60	57
Michigan	55	56	58	56	59	57
Minnesota			57	55	54	52
Mississippi			60	65	60	54
Missouri	62	64	57	57	57	50
Nebraska	60		61	64	66	59
New York	66	56	51	53		57
New York City	73	67	65	61	61	59
Oregon			53	69	64	57
Pennsylvania	64	64	61	58	60	56
Utah	65	66	62	73	67	58
Washington	58	61	62	64	64	61
Wisconsin	56	59	55	60	64	56
Wyoming	63	59	62	55	51	54

© 2024 McGovern M et al. *JAMA Network Open.*

eMethods

As well as the descriptive statistics shown in Table 1 in the main text, we assessed the association between experiencing homelessness and maternal and infant health by computing unadjusted and adjusted relative risk ratios. As outlined above, our exposure was defined as experiencing homelessness or having to sleep outside, in a car, or in a shelter, in the 12 months before the new baby was born. We considered the following outcomes in this analysis for the postpartum person: the month of pregnancy during which they had their first prenatal-checkup, whether they had high blood pressure during pregnancy, whether they had diabetes during pregnancy, and whether they had post postpartum depression, and for the infant: whether they were small for gestational age. The variables for month of first prenatal-checkup and small for gestational age are derived from birth certificate data that is linked to PRAMS. The exposure and other postpartum person outcomes are derived from the self-reported data. The month of pregnancy during which the postpartum person had their first prenatal-checkup is a count variable ranging from 1 (first month of pregnancy) to 9 (last month of pregnancy), while the other variables are dichotomous indictors. Small for gestational age is defined as a birthweight of less than 10th percentile for gestational age.

We computed relative risk ratios using modified Poisson regression models that include PRAMS weights and account for survey design.[3] Models for unadjusted relative risk ratios included only the exposure (an indicator for experience of homelessness) and the outcome. Models for adjusted relative risk ratios that accounted for observed potential confounders additionally included the following covariates that are shown in Table 1: infant is a singleton, infant sex, the postpartum person is married, postpartum person's place of residence (urban or rural), postpartum person's ethnicity, postpartum person's race, postpartum person's partner race, and postpartum person's age. For missing values on these covariates, we included an indicator variable in the regression for a missing category. These estimates are summarized in

© 2024 McGovern M et al. *JAMA Network Open.*

Figure 1 in the main text. In robustness checks, we found that results were not affected by the omission of urban/rural location of residence, infant sex, or plurality from the models.

© 2024 McGovern M et al. *JAMA Network Open.*

eReferences

1. Eliason EL, Agostino J, MacDougall H. Social Determinants and Perinatal Hardships During the COVID-19 Pandemic. *Journal of Women's Health*. 2024;33(3):371-378. doi:10.1089/jwh.2023.0290

2. Shulman HB, D'Angelo DV, Harrison L, Smith RA, Warner L. The Pregnancy Risk Assessment Monitoring System (PRAMS): Overview of Design and Methodology. *Am J Public Health*. 2018;108(10):1305-1313. doi:10.2105/AJPH.2018.304563

3. Zou G. A modified poisson regression approach to prospective studies with binary data. *American journal of epidemiology*. 2004;159(7):702-706.

© 2024 McGovern M et al. *JAMA Network Open.*
