# Supplementary material for: Homelessness and Maternal and Infant Health
Source: JAMA Netw Open. 2024 Nov 6;7(11):e2442596. doi: 10.1001/jamanetworkopen.2024.42596 (PMC11541637; doi:10.1001/jamanetworkopen.2024.42596)
Supplement: Supplement 2. — Data Sharing Statement [file jamanetwopen-e2442596-s002.pdf]

Data Sharing Statement

McGovern. Homelessness and Maternal and Infant Health. *JAMA Netw Open*. Published November 06, 2024. doi:10.1001/jamanetworkopen.2024.42596

Data
Data available: No

Additional Information
Explanation for why data not available: In this paper, we use data from the Pregnancy Risk Assessment Monitoring System (PRAMS), which are made available to researchers by the Centers for Disease Control and Prevention (https://www.cdc.gov/prams/php/data-research/index.html).
